# The role of fatty acids on ICSI outcomes: a prospective cohort study

**DOI:** 10.1186/s12944-016-0396-z

**Published:** 2017-01-21

**Authors:** Parvaneh Mirabi, Mohammad Javad Chaichi, Sedighe Esmaeilzadeh, Seyed Gholam Ali Jorsaraei, Ali Bijani, Mahjoobeh Ehsani, Seyedeh Fezzeh hashemi Karooee

**Affiliations:** 1 Infertility and Reproductive Health Research Center, Health Research Institute, Babol University of Medical Sciences, Babol, Iran; 2 Faculty of Chemistry, University of Mazandaran, Babolsar, Iran; 3 Social Determinants of Health Research Center, Health Research Institute, Babol University of Medical Sciences, Babol, Iran

**Keywords:** Fatty acids, ICSI, Follicular fluid, Oocyte quality, Embryo development

## Abstract

**Background:**

Our objective was to determine the effect of fatty acids (FAs) in serum and follicular fluid (FF) on fertilization and intracytoplasmic sperm injection (ICSI) outcomes.

**Methods:**

One hundred five women aged 18–38 years undergoing ICSI were recruited in this prospective cohort study. oocyte and emberyo quality was morphologically assessed. FAs in serum and FF were analyzed, using gas chromatography–mass spectrometry (GC-MS).

**Results:**

The mean number of mature oocytes was associated with serum levels of oleic acid (*r* = 0.58; *P* = 0.002). There were negative correlations between metaphase II oocytes and FF levels of stearic acid (*r* = −0.19; *P* = 0.04) and linolenic acid (*r* = −0.37; *P* = 0.004). According to the obtained Spearman’s correlation coefficients, serum levels of stearic, palmitoleic and tricosanoic acids were positively correlated with the percent of germinal vesicle (GV) stage oocyte.

The mean serum level of eicosapentaenoic acid was significantly higher in pregnant women than in non-pregnant patients (*P* = 0.006). Good quality embryos’ percentages were negatively correlated with the concentrations of palmitic acid (*r* = −0.22; *P* = 0.02).

After adjusting the effects of body mass index and age, total FAs were found to have a significant effect on the odds of having high-quality oocytes (percentage of oocytes > 80%; odds ratio =2.55; *P* = 0.054).

**Conclusion:**

Particular FAs affect oocyte maturation and implantation. Apparently, while higher FF levels of saturated FAs, especially palmitic and stearic acids, observed in some metabolic contexts have harmful effects on oocyte maturation and implantation, such effects can be counteracted and developmental competence can be enhanced (at least in vitro) by the presence of unsaturated FAs, e.g. oleic and eicosapentaenoic acids.

## Background

Fatty acids (FAs), commonly classified a saturated (SFA), monounsaturated (MUFA), and polyunsaturated (PUFA), play an important role in oocyte maturation and embryo development [1, 2]. While many studies have evaluated the effects of FAs on oocyte maturation and early embryo development in animals, little is known about the effects of FAs on human fertility [2–6]. According to previous animal and human studies, similar FAs are found in the FF and blood serum. However, follicular fluid levels of FFA are generally lower than those in the serum [7].

The most common FAs in animal and human FF are oleic, palmitic, and stearic acids [8] and palmitic, oleic, and linoleic acids, respectively [1]. Animal model studies have reported relations between SFAs, such as palmitic and stearic acids, and decreased rates of fertilization, cleavage, and blastocyst formation. These FAs inhibit granulosa and theca cell proliferation and induce apoptosis. On the contrary, arachidonic acid has a protective effect on granulosa cells [4, 8, 9]. Unsaturated FAs, such as oleic acid, enhanced blastocyst formation, oocyte maturation and embryo development [1, 9]. In fact, oleic acid can compensate for the negative effects of palmitic and stearic acids. Moreover, elevated levels of oleic acid do not have any negative effects and can even improve post-fertilization developmental competence [10]. In a study on pigs, the most common FAs found in immature pig oocytes were palmitic and oleic [11].

Linoleic acid and alpha-linoleic acid are two PUFAs, which can be both found in plants and plant oils, are particularly important in daily diet. Haggarty et al. detected significantly higher levels of linoleic acid in embryos which developed beyond the four-cell stage and concluded that this acid played a significant role in a number of key embryo development processes [12]. A correlation between linoleic acid and fertility rates was also documented in another study [13]. However, other studies argued that high levels of linoleic acid negatively affected oocyte maturation and developmental competence in animal models [9, 14]. It has also been found to exert a damaging effect on human reproductive system and decrease the chance of pregnancy after in vitro fertilization [15].

Since the majority of previous studies focused on animal models and reported controversial results about the effects of serum and follicular fluid FAs on oocyte maturation and post-fertilization developmental competence, this study evaluated associations between serum and follicular fluid concentrations of FAs and oocyte quality, embryo development, and pregnancy rate in women who underwent intracytoplasmic sperm injection (ICSI).

## Methods

### Patients

From March 2015 to July 2015 a total of 105 women, 22–38 years of age, who undergone ICSI and embryo transfer at Fatemeh Zahra Infertility and Reproductive Health Research Center, Babol University of medical sciences, Iran, were included in the study.

### Oocyte retrieval

All patients had a transvaginal ultrasound (TVS) on the third day of menstruation (5 MHz probe Fokuda, Japan) to rule out ovarian cysts. Following ovarian suppression by the subcutaneous injection of 0.1 mg gonadotropin releasing hormone analog (triptorelin, Diphereline, IpsenPharma Biotech, France) from the midluteal phase of the preceding cycle, controlled ovarian stimulation was started with a dose of 75–150 IU recombinant human follicle stimulating hormone (rFSH, 75 IU GONAL-f, Merck Serono, Germany) (HMG, Fostimon75 IU/Ampule IBSA, Switzerland) were given daily until the average diameter of the leading follicle reached 18–20 mm. Then Intramuscular HCG (Karma, Germany) at a dose of 10,000 IU was administered. Under ultrasound *g*uidance, oocyte retrieval was performed 36 h after HCG administration. Sample of venous blood was collected on the day of oocyte retrieval.

Collected oocytes according to their morphology, were classified into three grades; Metaphase II (MII), metaphase I (MI) and Germinal vesicles (GV) [16]. After removal of the oocyte*,* FF and coagulated blood were immediately centrifuged (1000 g, min) and stored at −80 °C until analysis*.*

Retrieved oocytes were fertilized by ICSI and cultured until the blastocyst stage. Embryos with, little or no fragmentation, and a zona pellucida that is not extremely thick or dark in appearance were classified as Grade A, embryos with equally-sized blastomeres, minor cyotplasmic fragmentation covering ≤ 10% of the embryo surface Grade-B and blastomeres of distinctly unequal size and moderate-to-significant cytoplasmic fragmentation covering >10% of the embryo surface Grade C.

A written consent was obtained from each patient for the use of FF and the study design was approved by the Ethical Review Committee of Babol University of Medical Sciences.

Clinical pregnancy was confirmed if gestational sac and fetal pole were visualized 7 weeks later.

### Extraction method

Serum and follicular fluid samples (1.5 ml each) were shaken on a platform vortexer for three minutes. After ward, 3 ml ice-cold acetone (Fluka, Buchs, Switzerland) were added to the samples to precipitate plasma proteins. The samples were then vortexed again for several seconds and maintained at −20 °C for 15 min. After the centrifugation of the precipitated proteins (Universal 2S Centrifuge, Hettich Lab Technology, Germany), the supernatant was separated and added with 3-ml aliquots of hexane (Fluka, Buchs, Switzerland) and water. The samples were then securely closed with Teflon-lined caps and shaken gently in a horizontal platform shaker for 5 min. Centrifugation was then performed to separate the solvent and the aqueous phases. The upper phase (hexane) was transferred into sterile tubes. A 0.25 ml aliquot of buffer, containing 0.2 M dibasic potassium phosphate with a pH of 9.0 and tri basic potassium phosphate (Sigma-Aldrich, USA) and 0.25 ml iodomethane (Fluka, Buchs, Switzerland) in dichloromethane (1:10 vol:vol) were added and the samples were vortexed for 5 min to form the free acid methyl esters (FAME). FAs of the samples were then separated and identified through capillary gas chromatography using an HB5column (dimensions: 30 m, 0.25 mm, and 0.25 μm) installed in a headspace gas chromatography/mass spectrometry device (Agilent technology GC 7890 and Mass 5975 detector).

Standard samples of FAs were obtained from Merck (Darmstadt, Germany) and the FAME standard (C4-C24) was purchased from Supelco (Sigma-Aldrich, USA).

## The experiment

### Reagents and materials

All the solutions were prepared using reagent-grade chemicals and deionized-distilled water. The standard FAME samples (oleic, stearic, palmitic, palmitoleic, myristic, arachidonic eicosapetaenoic (EPA), tricosanoic, docosahexaenoic (DHA) and linoleic methyl esters) were obtained from Supelco Inc. (Sigma-Aldrich, USA). The derivatization reagent was iodomethane (Sigma-Aldrich, USA). The phosphate buffer (pH = 9.0) was prepared by mixing 0.1 MNa3PO4 and 0.1 M Na2HPO4 (both from Sigma-Aldrich, USA) in water. Hexane and dichloromethane were obtained from Merck (Darmstadt, Germany).

### Apparatus

Quantitative GC-MS analysis was determined by using a GC: Agilent7890 and MS: Agilent5975c (with an HB5column). A model 220 Corning pH meter was used to carry out the pH measurements. Centrifugation was carried out by Hettich (Model Universal2S)

## Results

Of the 119 women initially considered for inclusion in the study, four had only one growing follicle and no oocytes to be retrieved and 10 had no chance of carrying a viable embryo. Therefore, 105 women were finally included in the study. Table 1 shows the baseline characteristics, basal hormone profile and reproductive outcomes of the participants. Correlation analysis revealed strong relationships between serum and follicular fluid levels of myristic acid (*r* = 0.63; *P* < 0.001), linolenic acid (*r* = 0.52; *P* = 0.001), and linoleic acid (*r* = 0.44; *P* = 0.003). However, the correlations between serum and follicular fluid levels of palmitoleic acid (*r* = 0.2; *P* = 0.03), EPA (*r* = 0.18; *P* = 0.06), DHA (*r* = 0.23; *P* = 0.02), and arachidonic acid (*r* = 0.22; *P* = 0.03) were weak. No significant correlations were found between serum and follicular fluid levels of palmitic acid (*r* = 0.12; *P* = 0.21), stearic acid (*r* = 0.12; *P* = 0.19), oleic acid (*r* = 0.06; *P* = 0.49), caproic acid (*r* = 0.12; *P* = 0.54) and tricosanoic acid (*r* = 0.02; *P* = 0.79).

**Table 1:** Clinical characteristic’s of sampled patients

	Mean ± SD
	n (%)
Age (year)	31 ± 6
BMI (kg/m^2^) ^a^	27.08 ± 4.2
Duration of infertility (year)	5.04 ± 4.27
Infertility	
Primary	81 (77.1%)
Secondary	24 (22.9%)
Cause of infertility	
Male factor	41 (39%)
Tubal Factor	6 (5.7%)
PCO	13 (12.5%)
Endometriosis	4 (3.8%)
Unexplained	31 (29.5%)
Both of Male & Female Factor	10 (9.5%)
Menstruation	
Regular	84 (80%)
Irregular	6 (5.7%)
Oligomenorrhea	15 (14.3%)
Semen characteristics	
Sperm count (10^6^/ml)	48.54 ± 39
Sperm Motility (%)	2.1 ± 1.01
FSH (mlu/ml)	7.5 ± 3.5
LH (mlu/ml)	7 ± 6.53
Prolactin (ng/ml)	23 ± 2.6
No. oocyte Oocyte quality	9 ± 5.6
MII	79% ± 30%
MI	9% ± 18%
GV	4% ± 13%
No. Embryo	4.38 ± 4%
No. embryos/No. oocytes	51% ± 27%
No. good quality embryos/No. embryo	85% ± 22%
No. good embryo/No. oocytes	43% ± 27%
Clinical pregnancy n (%)	29 (27.6%)

**Fig. 1:**
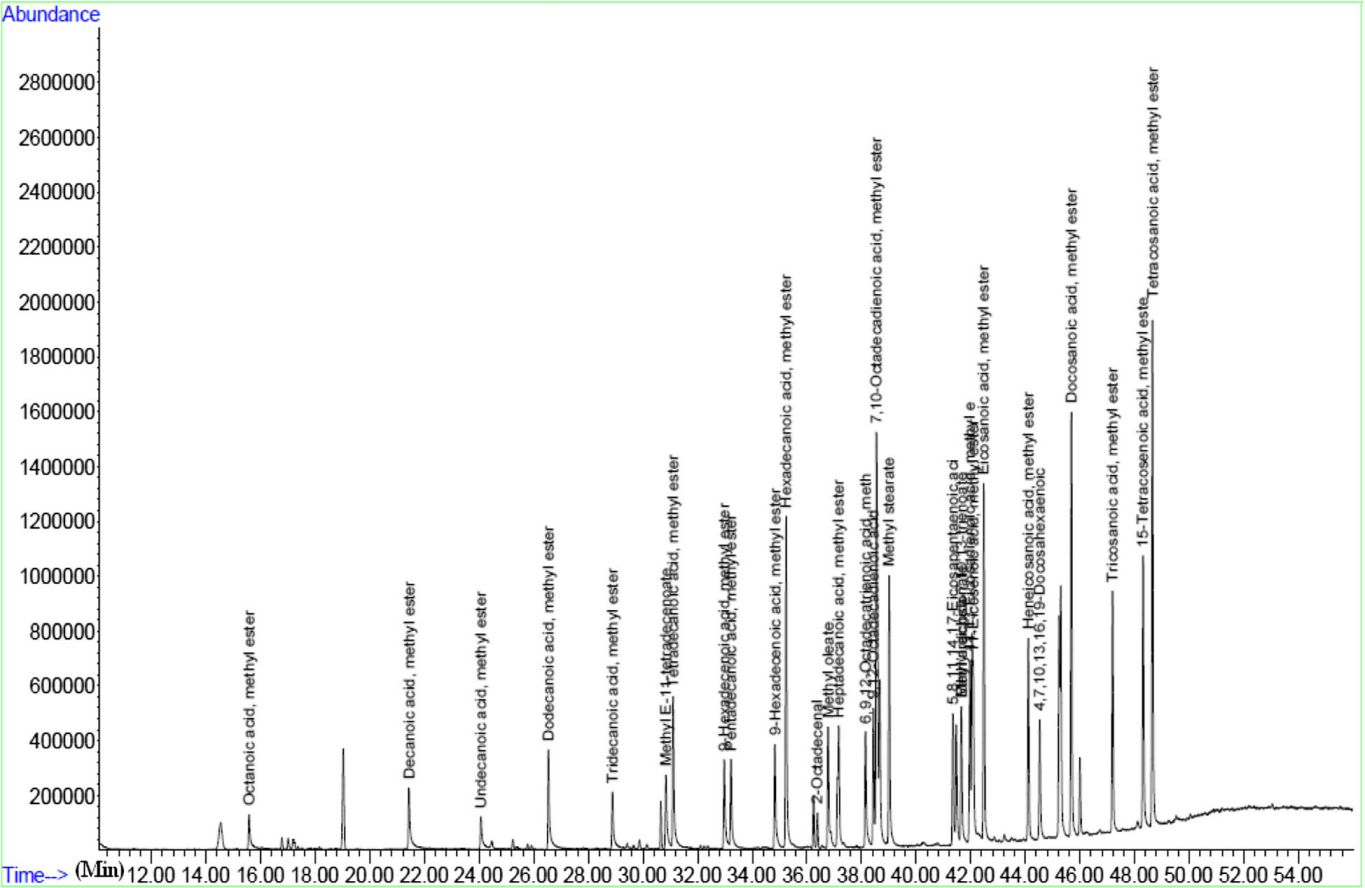
The GC/MS chromatogram of the fatty acid methyl esters

**Fig. 2:**
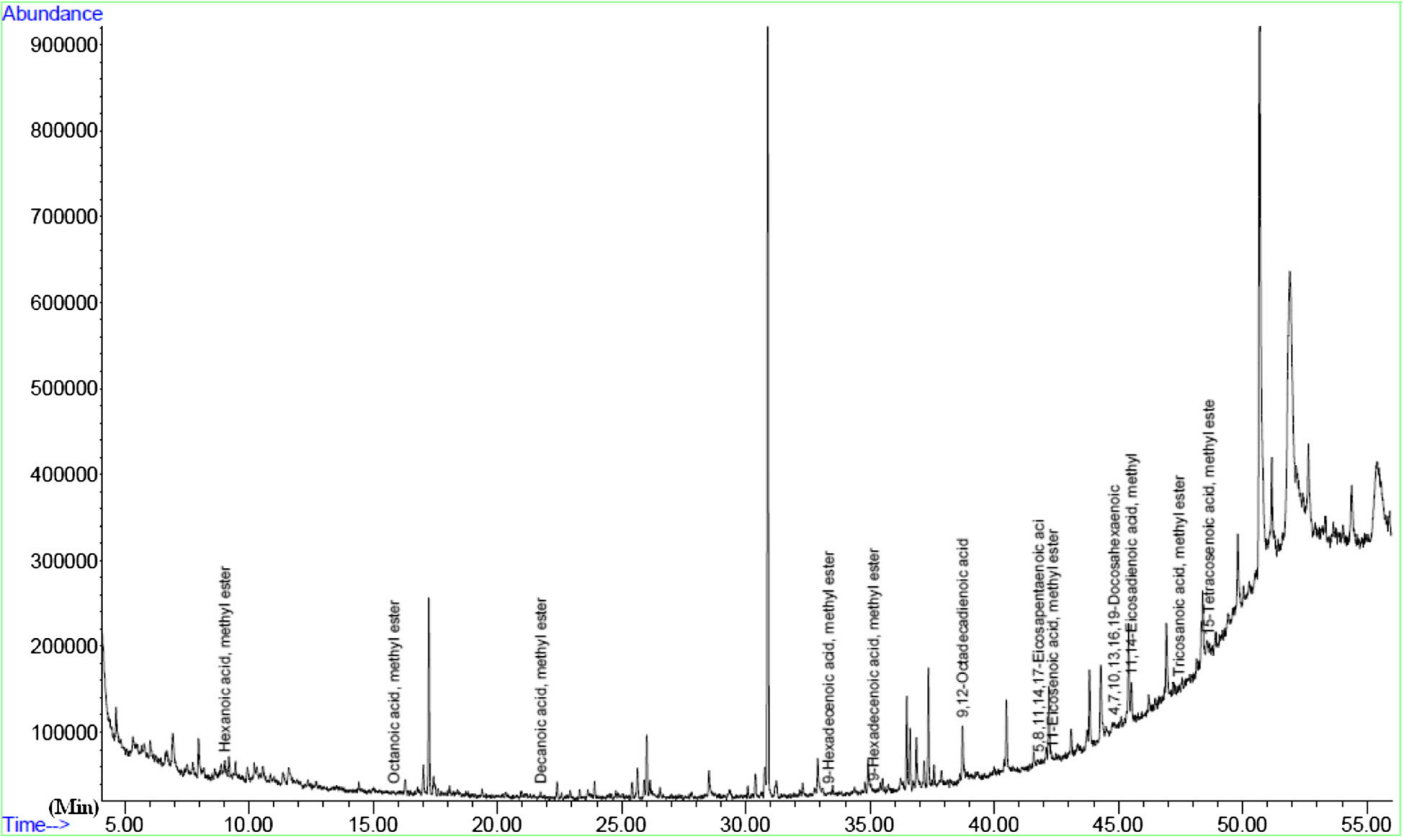
Typical output of GC/MS instrument separation of methyl esters substituted fatty acids in the follicular fluid sample

**Fig. 3:**
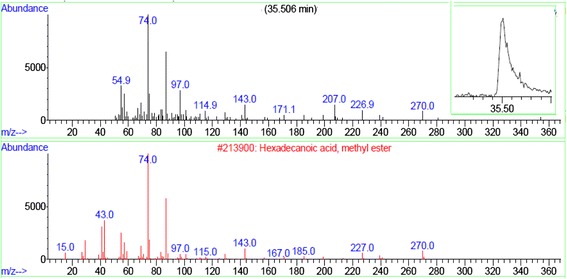
The output from a GC/MS instrument for the peak in the chromatogram. The upper curve corresponds to the mass spectra of the sample at the retention time of 35.506 min. The lower plot is the computer reconstructed mass spectra from the instrument library for hexadecanoic acid, methyl ester

Figures 1, 2, and 3 show the output from a GC/MS instrument for the peak in the chromatogram. As seen in Table 2, the mean number of mature oocytes was associated with serum levels of oleic acid (*r* = 0.58; *P* = 0.002). Moreover, there were negative correlations between metaphase II (MII) oocytes and follicular fluid levels of stearic acid (*r* = −0.19; *P* = 0.04) and linolenic acid (*r* = −0.37; *P* = 0.004). According to the obtained Spearman’s correlation coefficients, serum levels of palmitoleic and tricosanoic acids were positively correlated with the percent of germinal vesicle (GV) stage oocytes (Table 2).

**Table 2:** Assessment of follicular fluid and serum fatty acids with the outcome of pregnancy

	Fatty acid (ppm)	No. oocyte	MII	MI	GV	Good embryo
Follicular Fluid fatty acids	Palmitic (C16:0)	−0.4 (0.69)	−0.01 (0.89)	−0.003 (0.97)	0.07 (0.44)	0.08 (0.39)
	Palmitoleic (C16:1)	0.11 (0.24)	0.05 (0.61)	−0.06 (0.53)	0.02 (0.80)	0.04 (0.68)
	Stearic (C18:0)	0.08 (0.4)	**−0.19 (0.04)**	−0.08 (0.39)	0.10 (0.28)	0.03 (0.77)
	Myristic (C14:0)	−0.04 (0.64)	−0.04 (0.66)	0.03 (0.71)	0.07 (0.43)	0.02 (0.80)
	Oleic (C18:1)	−0.43 (0.66)	0 (0.98)	0.07 (0.45)	0.09 (0.32)	−0.02 (0.81)
	linolenic (C18:3)	−0.14 (0.27)	−**0.37 (0.004)**	0.04 (0.075)	0.12 (0.33)	−0.06 (0.63)
	Linoleic (C18:2)	−0.04 (0.65)	−0.01 (0.90)	−0.02 (0.81)	0.09 (0.35)	0.02 (0.81)
	Arachidonic (C20:4)	0.16 (0.13)	0.02 (0.81)	−0.03 (0.72)	0.05 (0.63)	−0.11 (0.33)
	Caproic (C6: 0)	−0.18 (0.35)	0.04 (0.81)	0.03 (0.87)	−0.09 (0.64)	0.16 (0.4)
	EPA (C20:5)	0.10 (0.30)	0.05 (0.61)	−0.03 (0.75)	−0.10 (0.30)	0.05 (0.56)
	Tricosanoic (C23:0)	0.04 (0.65)	0.05 (0.58)	−0.03 (0.76)	−0.04 (0.66)	0.05 (0.60)
	DHA (C22:6)	0.05 (0.64)	0 (0.93)	−0.05 (0.62)	0.13 (0.23)	−0.04 (0.70
Serum fatty acids	Palmitic (C16:0)	0.07 (0.45)	0.12 (0.23)	0.14 (0.142)	0.16 (0.88)	−0.009 (0.93)
	Palmitoleic (C16:1)	1.01 (0.67)	−0.14 (0.15)	0.02 (0.71)	**0.28 (0.005)**	−0.01 (0.90)
	Stearic (C18:0)	−0.09 (0.37)	−0.09 (0.35)	−0.05 (0.59)	**0.31 (0.002)**	0.02 (0.80)
	Myristic (C14:0)	−0.06 (0.54)	−0.02 (0.79)	0.10 (0.31)	0.06 (0.54)	0.03 (0.76)
	Oleic (C18:1)	**0.58 (0.002)**	0.07 (0.45)	−0.06 (0.53)	−0.04 (0.72)	0.07 (0.50)
	linolenic (C18:3)	−0.15 (0.25)	0.05 (0.68)	−0.04 (0.74)	0.13 (0.33)	0.01 (0.90)
	Linoleic (C18:2)	−0.04 (0.67)	0.13 (0.19)	−0.10 (0.29)	−0.06 (0.50)	0.11 (0.28)
	Arachidonic (C20:4)	−0.16 (0.88)	0.12 (0.26)	−0.10 (0.35)	−0.04 (0.65)	0.12 (0.26)
	Caproic (C6: 0)	−0.06 (0.42)	0.13 (0.49)	−0.008 (0.97)	−0.17 (0.40)	−1 (0.63)
	EPA (C20:5)	−0.02 (0.77)	0.01 (0.84)	0.04 (0.64)	−0.05 (0.59)	0.06 (0.54)
	Tricosanoic (C23:0)	0.01 (0.89)	−0.10 (0.31)	−0.003 (0.97)	**0.24 (0.014)**	−0.14 (0.16)
	DHA (C22:6)	−0.08 (0.43)	0.10 (0.35)	−0.10 (0.36)	−0.01 (0.91)	0.11 (0.32)

Values are reported as Correlation coefficient (*P*-value)

Table 3 compares the mean follicular fluid and serum concentrations of FAs in pregnant and non-pregnant women. Student’s *t*-test results showed a significant difference between follicular fluid concentration of palmitic acid in pregnant and non-pregnant women (*P* = 0.02). In addition, the mean serum level of EPA c acid was significantly higher in pregnant women than in non-pregnant patients (*P* = 0.006). Good quality embryos’ percentages (2B and 4A) were negatively correlated with the concentrations of palmitic (*r* = −0.22; *P* = 0.02).

**Table 3:** Assessment of follicular fluid and serum FFAs level in pregnant and non pregnant women

Fatty acid	Serum			Follicular fluid		
	Pregnancy positive	Pregnancy negative	*P*-value	Pregnancy positive	Pregnancy negative	*P*-value
	Mean ± SD	Mean ± SD		Mean ± SD	Mean ± SD	
Palmitoleic (C16:1)	29 ± 22.1	28.7 ± 22	0.96	42.3 ± 13.4	25.64 ± 2	0.35
Stearic (C18:0)	39.12 ± 23.3	24.12 ± 16.5	0.36	12 ± 8.5	19 ± 12.2	0.43
Myristic (C14:0)	4.72 ± 4	4.22 ± 4	0.54	3.02 ± 3.5	2.95 ± 3.3	0.92
Oleic (C18:1)	188.26 ± 83	31.58 ± 65	0.32	18.14 ± 25.3	27 ± 54.04	0.41
Palmitic (C16:0)	19 ± 47	34.44 ± 17.2	0.28	11 ± 9.3	25.38 ± 14	**0.02**
linolenic (C18:3)	6.33 ± 4.2	6 ± 4	0.87	5.59 ± 4	6 ± 6.50	0.87
Arachidonic (C20:4)	54.45 ± 16	44.58 ± 15	0.79	13.60 ± 48	14 ± 38.5	0.99
Linoleic (C18:2)	48.54 ± 14	42.44 ± 140	0.84	19 ± 45	30.08 ± 10	0.57
Caproic (C6: 0)	93.16 ± 13.6	56 ± 134	0.55	37.33 ± 85	31 ± 7.9	0.83
EPA (C20:5)	23.06 ± 18	15.68 ± 9	**0.006**	21 ± 2.6	18.37 ± 9	0.61
Tricosanoic (C23:0)	15.33 ± 6.01	14.42 ± 6	0.49	40 ± 131	18 ± 33.3	0.38
DHA (C22:6)	101.09 ± 26	107.5 ± 339	0.93	53.5 ± 135	78 ± 16	0.52

**Fig. 4:**
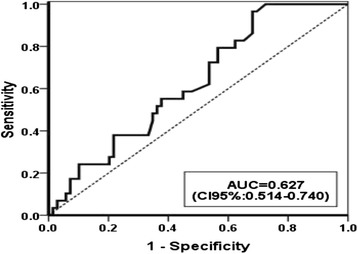
ROC curve for Total fatty acid in discriminating patients with poor quality oocytes from those with good quality

A receiver operating characteristic (ROC) curve analysis was conducted to determine the level of follicular fluid FA which best predicted reduced oocyte quality (defined as patient whose less than 80% of her oocytes were grade 3). AUC for total FA in detecting patients with low quality versus high quality oocytes was 0.62 (95% confidence interval: (0.51–0.74); *P* = 0.048; Fig. 4).

The optimal cut-off point for total FAs was 1.63 with a sensitivity and specificity of 55 and 62%, respectively. After adjusting the effects of body mass index (BMI) and age in logistic regression, total FAs (<1.63 and >1.63) were found to have a significant effect on the odds of having high-quality oocytes which was defined as patients with more than 80% of good oocytes; odds ratio =2.55; *P* = 0.054).

## Discussion

This study aimed to investigate the effects of serum and follicular fluid concentrations of FAs on the ICSI outcome. Since serum plays a major role in the composition of FF, metabolic changes in serum can alter the biochemical compounds of the FF. Follicle maturation is, hence, affected by blood metabolite concentrations. Several studies have shown that most serum FAs are reflected in the follicular fluid composition, but at lower levels [2, 17, 18].

The results of this study showed a positive correlation between FF and blood serum concentrations of PUFAs, such as linolenic and linoleic acids, which are essential FAs that cannot be synthesized by the body. On the other hand, no significant relationship was found between serum and follicular fluid concentrations of SFAs, including palmitic, stearic, and caproic acids. Likewise, Jungheim et al. could not establish a significant relationship between serum and FF concentrations of palmitic and stearic acids [19].

The pregnancy rate in this study, i.e. 27.6%, was lower than that reported in previous studies [7, 15]. ICSI failed in a number of our patients although they had high-quality oocytes and normal hysteroscopy results. Since serum levels of FAs are directly related to food intake and adiposity [20] the low success rate of ICSI might have been caused by the nutrition status and lifestyle of the patients (most patients in this study were actually overweight and their mean BMI was 27.08 ± 4.21 kg/m^2^). Another common cause of ICSI failure is reduced endometrial receptivity. The role of FAs as energy providers in decidualization has been shown in many studies. FAs and the β-oxidation pathway have been shown to be critical for oocyte growth and differentiation and embryo implantation [21, 22]. However, limited data is available about the relationship between FA metabolism and human embryo differentiation and implantation.

Most previous animal studies have evaluated bovine levels of FAs. Humans and Bovine usually ovulate a single competent oocyte during each cycle. Moreover, due to the similarities between human and bovine ovarian function and oocyte characteristics, the Bovine can be an excellent model for human reproductive system studies. According to animal studies, the early embryonic development is associated with higher complexity of FAs, especially an elevation in arachidonic acid levels [23, 24]. However, comparisons between human and animal oocytes showed the concentrations of oleic and linoleic acids in early stage human embryos to be respectively one-third and half those in animal oocytes. Therefore; further studies are required to directly examine the role of fat metabolism in the development of human embryos and the quality of embryos. In a human study, Haggarty et al. [12] Showed significantly higher levels of linoleic and oleic acids and a lower concentration of total saturated FAs in embryos that developed beyond the four-cell stage. They also found higher concentrations of arachidonic and DHA in the later stages of development.

These results are consistent with the data presented here which indicated not only positive correlations between oleic and EPA and oocyte maturation and implantation, but also a negative correlation between stearic acid and MII oocytes. We also detected significantly positive correlations between the concentrations of stearic, palmitoleic, and tricosanoic acids and immature (GV) oocytes. Although FAs are important in oocyte differentiation and embryo implantation, their high levels might have an adverse effect on the reproductive outcome. Previous animal model studies have demonstrated that the concentrations of FAs (i.e. SFAs, MUFAs, and PUFAs) in the follicular fluid and a fat-rich diet affected the developmental competence and fertilization potential of oocytes and thus embryo development. However, there have been contradictory results in this regard.

Several reports have shown that saturated FAs, such as palmitic and stearic acids, had an adverse effect on oocyte quality. Several in vitro and in vivo animal studies have documented the inhibitory effects of stearic and palmitic acids on early cleavage and number of oocytes reaching the blastocyst stage. They have reported that the capacity of cleaved zygotes to become blastocysts was lower in higher levels of stearic acid. In fact, high levels of stearic acid increased both amino acid metabolism and nuclear fragmentation which in turn caused higher apoptotic cell ratio and DNA damage [25–27]. Jungheim et al. concluded that moderate levels of palmitic acid decreased cell proliferation and the number of cells in the inner cell mass and increased the apoptosis of trophoblast stem cells [3]. Consistent results were reported in a study on human granulosa cells where palmitic and stearic acids impaired fertility by suppressing human granulosa cell survival and inducing apoptosis [28].

In contrast the findings of this study and previous research, some researchers highlighted the vital role and beneficial effects of palmitic acid on oocyte maturation [29, 30].

Our most interesting finding was the negative effect of linolenic acid on oocyte maturation. Although this finding is inconsistent with the results of some published studies [31, 32], it is in agreement with those of some others indicating the role of linolenic acid in inhibiting the development of the oocytes to the MII stage and cumulus cell expansion [33, 34].

Linolenic acid is an essential omega-3 FA obtained from foods such as vegetable oils, flaxseed, and soy seed. It is called “essential” because it is needed for cell growth and differentiation [15]. Despite the positive role of poly unsaturated fat intake in health status, we observed elevated linolenic acid levels to be strongly correlated with the number of oocytes retrieved, but not with the ICSI outcome. Therefore, this fatty acid could be considered as a marker of ovarian response, but not of oocyte or embryo quality.

Another important finding of this study was the significant positive correlation between serum oleic acid levels and number of retrieved oocytes. Oleic acid is a MUFA critical for oocyte maturation. It can overcome the adverse effects of palmitic and stearic acids.

We also found the mean concentration of SFAs (palmitic and stearic acids) to be higher (but not significantly) in women with polycystic ovary syndrome (PCOS). Approximately half of our participants with PCOS were overweight*.* Previous studies have argued that obesity and food intake might contribute to serum and follicular fluid FA concentrations. Therefore, all of patients with PCOS should be referred to a nutritionist before the ICSI procedure. Meanwhile, we did not detect any differences in follicular fluid and serum levels of FAs between patients with and without endometriosis. Since only 13 women with PCOS and four women with endometriosis participated in our study, studies with larger sample size are required to confirm these findings.

NIU et al. determined plasma and follicular fluid FA contents of 55 patients with PCOS and concluded that oocyte development, degree of embryo fragmentation, and pregnancy outcomes were positively correlated with the plasma and follicular fluid levels of stearic and oleic acids [35]. Khanaki et al. found that the mean level of stearic acid was significantly lower in patients with endometriosis. Furthermore, the ratio of EPA to arachidonic acid was correlated with the severity of endometriosis [36]. Jungheim et al. reported higher levels of follicular fluid FAs in patients with endometriosis but they concluded that oocyte quality was mostly affected by aberrations in FA metabolism or dietary FA intake rather than endometriosis. They suggested modifications in the types of consumed fats in these patients [37]. Another study showed an association between trans-unsaturated fat consumption and incidence of endometriosis [29]. Further studies with larger sample size are, hence, required to assess the correlations of serum and follicular fluid levels of FAs with diet and severity of endometriosis among infertile women.

A limitation of this study was not considering the nutritional habits and adiposity and values of oxidant and antioxidant enzymes of our participants.

The prospective design and comparing both serum and follicular fluid levels of FAs with ICSI outcome were the major strengths of our study.

The findings raise salient questions for future research. In fact, further studies are warranted to clarify whether or not changes in diet and body weight alter serum and follicular fluid levels of FAs and consequently affect the assisted reproductive outcomes. Moreover, researchers are recommended to examine whether the total FA content could be used as a marker for determining the number of mature oocytes and quality of embryos.

## Conclusion

Particular FAs affect oocyte maturation and implantation. Apparently, while higher follicular fluid levels of SFAs, especially palmitic and stearic acids, observed in some metabolic contexts have harmful effects on oocyte maturation and implantation, such effects can be counteracted and developmental competence can be enhanced (at least in vitro) by the presence of unsaturated FAs, e.g. oleic acid and eicosapentaenoic acids.

## Abbreviations

BMI: Body mass index
DHA: Docosahexaenoic
EPA: Eicosapentaenoic
ET: Embryo transfer
FAME: Fatty acid methyl ester
FAs: Fatty acids
FF: Follicular fluid
GC/MS: Gas chromatography–mass spectrometry
GV: Germinal vesicle
ICSI: Intracytoplasmic sperm injection
MI: Metaphase I
MII: Metaphase II
MUFA: Monounsaturated fatty acid
PCOS: Poly cystic ovarian syndrome
PUFA: Polyunsaturated fatty acid
SFA: Saturated fatty acid
TVS: Transvaginal ultrasound.
